# A Case Study on Improving Intensive Care Unit (ICU) Services Reliability: *By Using Process Failure Mode and Effects Analysis* (PFMEA)

**DOI:** 10.5539/gjhs.v8n9p207

**Published:** 2016-01-31

**Authors:** Taraneh Yousefinezhadi, Farnaz Attar Jannesar Nobari, Faranak Behzadi Goodari, Mohammad Arab

**Affiliations:** 1 School of Public Health, Tehran University of Medical Sciences, Tehran, Iran; 2 University Jean Moulin Lyon 3, Lyon, France

**Keywords:** Failure Mode and Effects Analysis (FMEA), Intensive Care Unit (ICU)

## Abstract

**Introduction:**

In any complex human system, human error is inevitable and shows that can’t be eliminated by blaming wrong doers. So with the aim of improving Intensive Care Units (ICU) reliability in hospitals, this research tries to identify and analyze ICU’s process failure modes at the point of systematic approach to errors.

**Methods:**

In this descriptive research, data was gathered qualitatively by observations, document reviews, and Focus Group Discussions (FGDs) with the process owners in two selected ICUs in Tehran in 2014. But, data analysis was quantitative, based on failures’ Risk Priority Number (RPN) at the base of Failure Modes and Effects Analysis (FMEA) method used. Besides, some causes of failures were analyzed by qualitative Eindhoven Classification Model (ECM).

**Results:**

Through FMEA methodology, 378 potential failure modes from 180 ICU activities in hospital A and 184 potential failures from 99 ICU activities in hospital B were identified and evaluated. Then with 90% reliability (RPN≥100), totally 18 failures in hospital A and 42 ones in hospital B were identified as non-acceptable risks and then their causes were analyzed by ECM.

**Conclusions:**

Applying of modified PFMEA for improving two selected ICUs’ processes reliability in two different kinds of hospitals shows that this method empowers staff to identify, evaluate, prioritize and analyze all potential failure modes and also make them eager to identify their causes, recommend corrective actions and even participate in improving process without feeling blamed by top management. Moreover, by combining FMEA and ECM, team members can easily identify failure causes at the point of health care perspectives.

## 1. Introduction

In any complex human system, human error is inevitable. In acute care systems, some epidemiologic studies estimated that more than 1.3 million people suffer from unintended injuries each year in the United States (Duwe, Fuchs, & Hansen-Flaschen, 2005), while these errors’ frequency can be even increased in a more complicated health care system such as an Intensive Care Unit (ICU).

In the past, errors in any system and organization such as a healthcare system or a hospital were usually known as a “human error” (Duwe et al., 2005). There are two different approaches to study errors: person approach and system approach. In person approach, health care managers focus on wrong doers and mostly blame hospital staff for their forgetfulness, inattention and all other failures. But in system approach, most medical errors are recognized as a systematic one that can be prevented by making the workplace safe and reliable. In this approach, managers try to lead the health care staff to mitigate errors or their effects (Reason, 2000). According to the emerging of systematic approach to manage medical errors (Crane, & Crane, 2006), Failure Mode and Effects analysis (FMEA) is a good systematic technique, which prospectively identifies, evaluates, prioritizes and eliminates potential failure modes and effects in order to improve the safety, reliability, and quality of products and/or processes (Srivastava, & Mondal, 2014; Abbasgholizadeh Rahimi, Jamshidi, Ait-Kadi, & Ruiz, 2013). This method enables users to continually improve their quality and reliability of products and processes and also increase their customers satisfaction (Chen, & Ko, 2009; Karim, Smith, & Halgamuge, 2008). In fact, FMEA attempt to predict how and where systems might fail and then how it can change the system condition to the safer and more reliable condition to prevent unacceptable failures from occurring by fallible human or at least their effects from reaching to customers (Abbasgholizadeh Rahimi et al., 2013; Reason, 2000).

Using FMEA exactly goes back to 1949 when the American Army has confidentially evaluated failures of their system and equipment (Braaksma, Meesters, Klingenberg, & Hicks, 2012). Then, NASA has proposed FMEA methodology more open to public and used it for improving their requirement reliability in 1963 (Sharma, D. Kumar, & P. Kumar, 2007a). Thereafter, FMEA became well-known in automotive industries such as Ford Motor Company, Chrysler Corporation and General Motors Corporation especially as a requirement of ISO/TS 16949 (QS9000); World Automotive Standard (Estorilio, & Posso, 2010) and even it spread in other industries through some standards such as MIL-STD 1629A (used in the United States military), IEC 60812, BS EN 60812, and the SAE-J1739 standard (Braaksma et al., 2012) (Crysler Corporation, Ford Motor Company, 1995).

From then on, FMEA has been used in many production industries for more than 40 years such as nuclear power, aerospace, automotive, chemicals, electronics and food companies (Silvestri, De Felice, & Petrillo, 2012; Chuang, 2010; Ookalkar, Joshi, & Ookalkar, 2009). Nowadays, FMEA is a simple and powerful tool for improving safety and reliability of any system (Sharma, D. Kumar, & P. Kumar, 2007b). In comparison to well-known FMEA in many industries, FMEA is a little new concept in healthcare systems. The introduction of this method in healthcare systems goes back to Joint Commission on Accreditation of Healthcare Organizations (JCAHO) efforts to modify this method for health care systems in the name of Health Care FMEA or HFMEA in 2002 (Fibuch, & Ahmed, 2014). Afterwards, FMEA has been modified and applied across all hospital processes such as in a trauma, IV drug administration dialysis process, patient medical records, emergency department, ICUs, chemotherapy, pharmacy, etc. (Fibuch, & Ahmed, 2014; Ookalkar et al., 2009). Besides, the JCAHO has required all acute care units to use FMEA regularly to reduce medical errors (Chiozza, & Ponzetti, 2009).

According to literature reviews on FMEA study, this method follows some defined processes which can be different from 4 to 10 steps. Following the nine- step FMEA process can further explain the details:

1) - Select a problematic product/process and create a multidisciplinary team
2) - Define all functions of product/process
3) - Define potential failure modes for each function
4) - Determine the potential effects of each failure and evaluate the Severity (S) score
5) - Determine the potential causes of each failure and evaluate the Occurrence (O) score
6) - Determine the current controls for each failure and evaluate the Detectability (D) score
7) - Calculate all failures in Risk Priority Numbers (RPNs) and then prioritize all failures in a descending order
8) - Identify failure causes for high risk failures, recommend corrective actions and then implement them
9) - Recalculate new RPNs for high risk failures which then need corrective actions (Bradley, & Guerrero, 2011) (Chuang, 2010)(Ookalkar et al., 2009)

**Figure 1:**
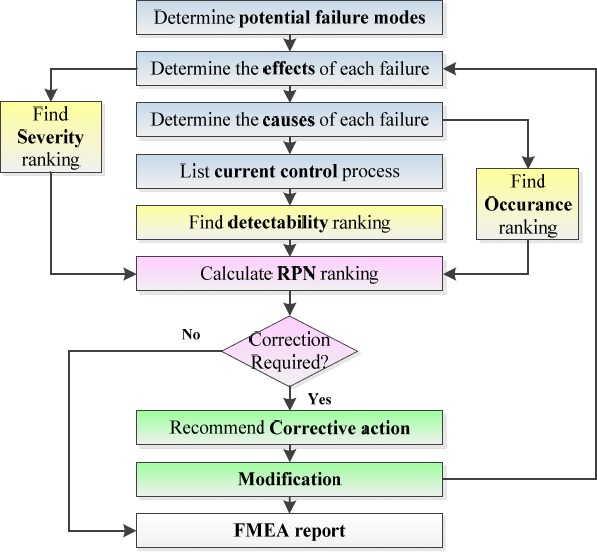
FMEA Process (Abbasgholizadeh Rahimi et al., 2013)

All steps of FMEA process can be observed in Figure 1.

It should be noted that according to FMEA objectives to improve the reliability of both the product and process, there are two types of FMEA: Design FMEA (DFMEA) and Process FMEA (PFMEA) (Segismundo, & Miguel, 2008).

The aim of this research was to anlyze the reliability of two selected ICU’s processes in Tehran through the proactive and systematic approach of PFMEA by identifying, evaluating, prioritizing and then analysing its potential failure modes. The prospective and systematic approach of FMEA was used to predict where ICU’s processes might fail and how we can change its condition to the more reliable level to prevent unaccepted failures before thier effects reach patients or systems.

Compared to the nine-step FMEA process, this FMEA study steps were as follows:

1) Process study
2) Failure identification
3) Failure evaluation
4) Failure prioritizing
5) Failure analysis

Besides, Eindhoven Classification Model (ECM) was applyied for identifying and classifying all the causes of determined high risk failures which let the FMEA team analyze failure causes more easily. To know more about ECM, this classification model has been proposed for medical errors’ Root Cause Analysis (RCA) by Eindhoven University of Technology. Errors in this model have been categorized in three main categories and twenty subcategories:

A) Errors related to *latent condition* which is divided into *technical* and *organizational*. Technical subcategories are external (T-EX), design (TD), construction (TC), and materials (TM), and organizational subcategories include external (O-EX), transfer of knowledge (OK), protocol (OP), management priorities (OM), and culture (OC).

B) *Active errors* or *human* category which includes four subcategories: external (H-EX), knowledge-based behavior (HKK), rule-based behavior (such as qualifications (HRQ), coordination (HRC), verification (HRV), intervention (HRI), and monitoring (HRM)), and skill-based behavior (such as slips (HSS) and tripping (HST)).

C) Other factors which cannot be classified in latent or active ones like the patient related factor (PRF) and unclassifiable (X) (Harmsen et al., 2010) (van Vuuren, Shea, & van der Schaaf, 1997).

## 2. Methods

### 2.1 Type of Research

This research is a descriptive one that defines and analyzes failures of two ICU’s processes through FMEA methodology in a prospective way.

Furthermore, this is a qualitative case study, in which the research participants as FMEA team members were selected purposefully from two studied ICU’s in Tehran based-on their skills and knowledge. In fact, before collecting data in this research, FMEA team members were selected for each two selected ICUs and then educated by researchers.

### 2.2 Data Collection Tools

According to the FMEA qualitative approach, research data was collected through observation and Focus Discussion Group (FDG) with FMEA team members. The FMEA team members’ positions were varied from chief nursing (matron), chief nursing assistant, educational nurse supervisor, clinical nurse supervisor, ICU’s head nurse, ICU’s bedside nurse to quality improvement and patient safety experts (See Appendix A for details). It should be noted that the FMEA team meetings were held weekly in selected ICU’s including 15 sessions in one of Tehran nongovernmental hospitals (A) and 10 sessions in one governmental hospital (B).

### 2.3 Data Collection Phases

This research data collection had five phases:

#### 2.3.1 Process Study Phase

ICU processes were selected, and then they were drawn into flow charts and defined in detailed activities within the FMEA team sessions in each ICU.

#### 2.3.2 Failure Identification Phase

Potential failure modes and effects were defined for each small detailed ICU activity and listed in the FMEA worksheets.

#### 2.3.3 Failure Evaluation Phase

All those failures were scored by FMEA team members based on three criteria: Severity (S), Occurrence (O) and Detectability (D); all from 1 to 10 scores which has been redefined by FMEA team according to studied process characteristics (See Table 1). Then, RPNs for each failure modes were calculated by multiplying all three criteria scores together (H.-C. Liu, L. Liu, & N. Liu, 2013; Sawhney, Subburaman, Sonntag, Rao, & Capizzi, 2010; Chin, Wang, Poon, & Yang, 2009).

**Table 1:** Severity, Occurrence and Detectability Score table used in selected ICUs

Score	S	O	D
10	Dangerous	More than one time along one nursing shift (8 hours)	Absolute Uncertainty
	without warning		10%>
9	Dangerous	One time along one day	Very Remote
	with warning		10%-20%
8	Very high	One time along 3 days	Remote
	and irretrievable		20%-30%
7	High	One time along one week	Very Low
	and retrievable		30%-40%
6	Moderate	One time along one month	Low
	and retrievable		40%-50%
5	Low	One time along 3 months	Moderate
	with obvious effect		50%-60%
4	Very low	One time along 8 months	Moderately High
	with less obvious effect		60%-70%
3	Minor	One time along 2 years	High
	with obvious effect		70%-80%
2	Very minor	One time along 6 years	Very High
	with less obvious effect		80%-90%
1	No effect	More than one time along 6 years	Almost Certain
			90%>

#### 2.3.4 Failure Prioritizing Phase

All evaluated failures were sorted based on RPNs (Liu et al., 2013; Chin, Wang, Ka Kwai Poon, Yang, & Poon, 2009). Then, by assigning the reliability bottom line for RPNs, high risk failures or not accepted failure modes were determined.

#### 2.3.5 Failure Analysis Phase

Finally, causes for each of not accepted failures were defined, classified, analyzed and then corrective actions were recommended for them by focus groups of FMEA team. Fishbone diagram (for defining causes) and Eindhoven Classification Model (ECM) (for classify them), were also used in this phase.

### 2.4 Data Analysis

In accordance to used FMEA method, data were analyzed quantitatively based on obtained RPNs which are 1<RPN<1000 in this case. In fact, all failures were prioritized at the base of RPN. Then, according to FMEA team decision making to improve ICU process reliability up to 90%, failures with RPN≥100 were listed as the not accepted failures and some their causes were analyzed by qualitative Eindhoven Classification Model (ECM) in the final step.

## 3. Results

### 3.1 Process Study Phase

FMEA team has selected common 9 ICU processes in both ICUs included: “Patient Delivery Process from ER to ICU”, “Patient Delivery Process from ward to ICU”, “Inpatient Administration Process”, “Taking Medication Process”, “Pressure Ulcer Caring Process”, “Endotracheal/Tracheal Intubation Process”, “Suction Process”, “Physiotherapy Process” and “Portable Radiology Process”.

Moreover, another 6 processes which were selected only in hospital A included: “Patient Delivery Process from ward to ICU”, “Mechanical Ventilation Providing Process”, “Patient Nasogastric Tube (NGT) Insertion Process”, “Chest Drain Insertion Process”, “Narcotic Injection Process” and “Death Certification and Corpse Protocol Process”.

Also, FMEA team has defined 180 activities for 15 ICU processes in hospital A and 99 activities for 9 ICU processes in hospital B in details.

### 3.2 Failure Identification Phase

The FMEA team has defined and listed all potential failure modes and effects for each detailed ICU activities into the FMEA worksheets. In this phase, 378 potential failure modes from 180 ICU tasks (or 15 processes) in hospital A and 184 potential failures from 99 ICU tasks (or 9 processes) in hospital B were identified.

### 3.3 Failure Evaluation Phase

The FMEA team members put three scores for each identified failures by focus groups according to Table 1. Then by multiplying these three criteria of each failure together, all failures’ RPNs were obtained.

### 3.4 Failure Prioritizing Phase

Researchers sorted all determined failures based on RPNs from high to low. Then FMEA team decided to put 90% reliability for failures (or failure with RPN≥100) in order to make corrections. Therefore, totally 18 failures in hospital A and 42 ones in hospital B were determined as non-acceptable failures. Top 10 not accepted failures are shown for both ICUs as an example (See Table 2).

**Table 2:** Top 10 non-accepted failure modes of two studied ICUs

Priority	Hospital A				Hospital B			
	Process	Activity	Failure Mode	RPN	Process	Activity	Failure Mode	RPN
1	Suction Process	Wash and scrub hands	Insufficient/imperfect hand wash and/or scrub by nurse	163	Suction Process	Wash and scrub hands	Insufficient/imperfect hand wash and/or scrub by nurse	480
2	Patient Delivery Process from Operating Room (OR) to ICU	Keep on patient caring in Recovery ward till ICU’s bed become ready to occupy	Non-consideration of patient caring requirements	158	Portable Radiography Process	View radiograph of the patient at PACS system by physician	Not viewing patient radiography and leave it to another physician	336
3	Suction Process	Check patient respiratory system and diagnose whether he/she needs suction or not at the point of nurse	Wrong diagnose for patient suction need	126	Endotracheal/Tracheal Intubation Process	Suck fluid from patient’s throat and mouth by physician and nurse	Imperfect/not sterile intubation	315
4	Pressure Ulcer Caring Process	Change patient position on bed	Not changing patient position on bed	125	Suction Process	Wash and scrub hands	Not wash and/or scrub hands by nurse	280
5	Inpatient Administration Process	Daily visit of patient by physician	Delay in daily visit	114	Taking Medication Process	Inform pharmacy to prepare medicine and wait (In the morning shift)	Delay in informing to ICU about deleting medicine on the system	252
6	Narcotic Injection Process	Register/Record physician order of narcotic for patient	Delay in recording ordered narcotic for patient	112	Taking Medication Process	Inject/give the medicine to patient according of ordered dose and duration by nurse	Wrong medication (dose, time and duration)	245
7	Taking Medication Process	Inject/give the medicine to patient according of ordered dose and duration by nurse	Wrong medication (dose, time and duration)	110	Physiotherapy Process	Come and make patient implement passive movements, exercises and educate him/her active movements by physiotherapist	Insufficient/imperfect education	240
8	Chest Drain Insertion Process	Use the folding screen or draw the curtain to keep patient privacy	Not using folding screen or curtain	110	Taking Medication Process	Deliver Pharmaceutical drug/Medicine to the nurse	Delay in deliver Pharmaceutical drug/Medicine because of finishing it	240
9	Corpse Protocol	Issuance death certification by physician	Delay in issuance of death certification	109	Taking Medication Process	Inject/give the medicine to patient according of ordered dose and duration by nurse	Not inject medicine to patient on time	240
10	Portable Radiography Process	Call with Radiology ward and arrange radiologist to ICU	Not responding to ICU radiography request	109	Physiotherapy Process	Register/Record physiotherapy order by physician	Not register/record physiotherapy order by physician	216

### 3.5 Failure Analysis Phase

The FMEA team was classified and defined non-acceptable failure causes and then suggested to make corrections for them according to Eindhoven Classification Model (ECM) and in the FMEA method framework (See Table 3).

**Table 3:** Sample of FMEA worksheet for 5 non-accepted failure modes in in two studied ICUs

Priority	Hospital A			

	Failure Mode	RPN	Causes	Corrective Actions Strategy
1	Insufficient/imperfect hand wash and/or scrub by nurse	163	*-Technical:*	Occurrence reduction strategy
			TM: Materials (1.not qualified antiseptic hand rub/sanitizer for hands’ skin with bad odor 2. lack of hand tissue to dry hands)	
			*- Organizational:*	
			OC: Culture (inappropriate culture and non-observance of hand wash and/or scrub necessity at the point of health care personnel)	
			OK: Knowledge Transfer (inadequate knowledge transferred to all new or inexperienced staff)	
			*-Human:*	
			HRM: Rule-based behavior- Monitoring (not systematic monitoring system of nurses in this issues)	
2	Non-consideration of patient caring requirements	158	*- Technical:*	Occurrence reduction strategy and detectability increase strategy
			TC: Construction (inappropriate physical construction of this ward which make difficulties in traffic ward and considering patient caring requirements)	
			TM: Materials (not enough recovery equipment such as pulse oximeter and patient monitors)	
			*- Organizational:*	
			OC: Culture (lack of continuity approach to patient care process by Operating Room(OR) nurses while they often focused on just delivering patients fast to ICUs)	
			*- Human:*	
			H-EX: External (heavy workload of nurses in this recovery room and the large number of surgeries)	
			HKK: Knowledge-based Behavior (lack of information of patient injected drug in recovery room given to the next nurse)	
			HRC: Rule-based behavior- Coordination (shift change in recovery room)	
			HRV: Rule-based behavior- Verification (excessive caution of physician or excessive tendency to improve patient care qualities that result in transferring patient into ICU regardless of the real need for ICU bed)		
3	Wrong diagnose for patient suction need	126	*- Human:*	Occurrence reduction strategy and detectability increase strategy
			H-EX: External (heavy workload of nurses in this ICU)	
			HKK: Knowledge-based Behavior and	
			HRQ: Rule-based behavior- Qualifications and HSS: Skill-based behavior- Slips	
			(novice nurse and/or alternative nurse)	
			HRV: Rule-based behavior- Verification (wrong patient triage and the ambiguous respiratory statues of patients who are delivered from Emergency Room (ER) or Operating Room (OR))	
			HRM: Rule-based behavior- Monitoring (changing shift of nurses)	
4	Not changing patient position on bed	125	*- Technical:*	Occurrence reduction strategy
			TM: Materials (not using from wavy mattress)	
			*- Organizational:*	
			OM: Management Priorities (inadequate nurse’s aide personnel which is a result of national limitation for engaging of nurse’s aide personnel from hospitals or management priorities and decision makings)	
			*- Human:*	
			HRQ: Rule-based behavior- Qualifications and HSS: Skill-based behavior- Slips	
			(Pulling patient on his/her bed instead of lifting up)	
			*- Other factors:*	
			X: Unclassifiable (limitation on changing patient position because of fracture of bone or so on)	
5	Delay in daily visit	114	*- Organizational:*	Occurrence reduction strategy
			OP: Protocol (for per case patients, other physician can’t visit patient until patient responsible physician apply)	
			OC: Culture (inappropriate organizational culture while 1.physicians tends to schedule his/her on-call day in ICU at the same time of his/her clinic day result in not presence of on-call physician in hospital clinic while he/she must be in ICU 2. (physician tendency to visit all patients at the end of his/her shift time)	
			*- Human:*	
			H-EX: External (1. not presence of responsible physician 2. heavy workload of nurses in this ICU)	
			HRV: Rule-based behavior- Verification	
			HRI: Rule-based behavior- Intervention	

**Priority**	**Hospital B**			

	**Failure Mode**	**RPN**	**Causes**	**Corrective Actions Strategy**

1	Insufficient/imperfect hand wash and/or scrub by nurse	480	*-Technical:*	Occurrence reduction strategy
			TM: Materials (1.not qualified antiseptic hand rub/sanitizer for hands’ skin with bad odor 2. lack of hand tissue to dry hands)	
			*- Organizational:*	
			OC: Culture (inappropriate culture and non-observance of hand wash and/or scrub necessity at the point of health care personnel)	
			OK: Knowledge Transfer (inadequate knowledge transferred to all new or inexperienced staff for example: train to all nurses that using latex gloves does not violate the requirements of hand wash and train them to disinfect their hands before and after using it)	
			*- Human:*	
			EX-H: External (lack of nurse time in the case of having critical patient or consecutiveness of taking medication to patient)	
			HRM: Rule-based behavior- Monitoring (not systematic monitoring system of nurses in this issues)	
			*- Other factor*	
PRF: Patient related factor (critical condition of patient)
2	Not viewing patient radiography and leave it to another physician	336	*- Technical:*	Occurrence reduction strategy and detectability increase strategy
			TD: Design (PACS system can’t prevent from this failure by alarming or informing to related physician and bedside nurse)	
			*- Organizational:*	
			OK: Knowledge Transfer (not informing to the next physician by related physician and bedside nurse)	
			*- Human:*	
			H-EX: External (heavy workload of physicians and nurses in this ICU)	

3	Imperfect/not sterile intubation	315	*- Technical:*	Occurrence reduction strategy
			TM: Materials (1. not enough sterile gloves and requirements 2. suction with not sterile gloves)	
			*- Organizational:*	
			OC: Culture (inappropriate culture)	
			*- Human:*	
			HRI: Rule-based behavior- Intervention (rapid turnover of medical staff)	
			HRM: Rule-based behavior- Monitoring (not systematic monitoring system of nurses in this issues)	
			HSS: Skill-based behavior- Slips(suction with high speed)	

4	Not wash and/or scrub hands by nurse	280	*- Technical:*	Occurrence reduction strategy
			TM: Materials (1.not qualified antiseptic hand rub/sanitizer for hands’ skin with bad odor 2. lack of hand tissue to dry hands)	
			*- Organizational:*	
			OC: Culture (inappropriate culture and non-observance of hand wash and/or scrub necessity at the point of health care personnel)	
			*- Human:*	
			HKK: Knowledge-based Behavior (not knowing that using latex gloves does not violate the requirements of hand wash and train them to disinfect their hands before and after using it)	
			HRI: Rule-based behavior- Intervention (busy nurse because of having critical patients and/or sequence of consecutive medication to patient or so on)	

5	Delay in informing to ICU about deleting medicine on the system	252	*- Organizational:*	Occurrence reduction strategy and detectability increase strategy and severity reduction strategy
			OK: Knowledge Transfer (not suitable informing system between drugstores and hospital ward)	
			OM: Management Priorities (not supply hospital with some needed drugs)	
			OC: Culture	
			*- Human:*	
			HRC: Rule-based behavior- Coordination (problem in cross sectional relationship and information system in hospital)	
			HRI: Rule-based behavior- Intervention (changing shift of nurses)	

## 4. Discussion

Intensive Care Units or ICU’s have a critical and data-rich environment considering their unconscious patients (with often unstable and crucial condition and need of immediate and timely medications), its high-tech and complex equipment (such as monitoring and drug injection equipment) and their usually high trained and multi-task clinicians (Hains, Georgiou, & Westbrook, 2012). Meanwhile, medical errors in ICU’s will not be far-fetched and should be prevented by applying some proactive approaches. FMEA is one of the proactive methods which are used for preventing errors, ensuring patient’s safety and improving of medical processes. Therefore, this PFMEA study was conducted with the aim of determining ICU’s process failures, causes and recommending corrective actions for eliminating and controlling them.

The results of this research in hospital A show that from 378 identified potential failure modes, 180 ICU activities and 15 processes, only 18 failures were listed as the not accepted failure modes with 90% reliability. The top 10 high risk failures were respectively related to “Suction Process”, “Patient Delivery Process from Operating Room (OR) to ICU”, “Pressure Ulcer Caring Process”, “Inpatient Administration Process”, “Narcotic Injection Process”, “Taking Medication Process”, “Chest Drain Insertion Process”, “Death Certification and Corpse Protocol Process” and “Portable Radiology Process”. In hospital B, results have been presented that from 184 failure modes 99 ICU activities and 9 processes, 42 failures were identified as the non-acceptable failures with 90 % reliability whose top 10 not accepted failures were respectively related to “Suction Process”, “Portable Radiology Process”, “Endotracheal/Tracheal Intubation Process”, “Taking Medication Process” and “Physiotherapy Process”. For identifying all root causes of not accepted failures in both studied ICU’s, Eindhoven Classification Model (ECM) was applied and all causes were classified into four main groups: technical, organizational, human and other factors.

The comparison of failure frequency in both ICU’s indicates that potential failure modes in hospital A (378 failures) were more than failures in hospital B (about 184), while the RPN’s of failure modes in hospital B were so higher than hospital A. At first glance, differences of failure frequencies in both hospitals can be the result of the number of ICU activities or processes which were studied. Obviously, 180 ICU activities within 15 processes in hospital A were more than 99 ICU activities within 9 processes in hospital B. But viewed with more scrutiny, these differences can be the result of other factors such as hospital type, characteristics and participation rates of team members, daily patient admission and so on. As mentioned before, hospital A is nongovernmental (semi-private) hospital while hospital B is a governmental and teaching hospital. Besides, it is observed that FMEA team in hospital A was more active in brainstorming potential failure modes which can be the result of their characteristics and having less workload or more time to participate in team sessions. Probably the more not accepted failure modes in hospital B (42 failures in comparison to 18 ones) and also higher RPN’s of failure modes (the highest is 480 compared to 163) can be the result of more patient admission or teaching entity of the hospital in hospital B, but more research is should be conducted to draw conclusions in this way. In a similar study, *Asefzadeh and et al*. have identified 48 clinical failures while respectively from the highest to the lowest were “ventilator’s alarm malfunction (no alarm)” with the score 288 and “not washing the NG-Tube” with the score 8. Moreover, they have represented that actions related to training and improving clinical cares and also proper shift scheduling are important factors in reducing potential failures and can result in clinical risk management and the improvement of patient safety (Asefzadeh, Yarmohammadian, Nikpey, & Atighechian, 2013).

### 4.1 The Most Top Failures

Comparing the overall results of not accepted failure modes in the selected ICU’s have shown that the most high risk failure is the same in both hospitals. In other words, the highest risk failure in both ICU’s is the “Insufficient and/or imperfect hand wash and/or scrub by nurse” in “Suction Process”. Before discussing this failure, it should be defined that “suction process” is a procedure which removes substances from the trachea, pharynx, nose or mouth either naturally by nose or mouth or with artificial tubing such as endotracheal tube, tracheostomy tube and nasal or oral airway (Overend et al., 2009). Hand washing is always considered as the most important intervention of preventing nosocomial infection that can be transmitted mostly by the healthcare workers’ hands. In addition, hand hygiene can be considered as a sole measure for an effective infection reduction when other factors in infection control are inadequate, such as the environmental hygiene, crowding and education level of staff. Despite the simplicity of hand hygiene techniques, the interdependence of other factors makes hand hygiene behavior more complex (McLaws, Farahangiz, Palenik, & Askarian, 2015; Akyol, Ulusoy, & Özen, 2006).

A lot of research has been conducted on hand hygiene field. Most of the medical literature of hand hygiene error concerns healthcare institutions in developed countries whereas the high threat of infectious diseases are more in developing countries and also basic sanitation accessibility is often limited or nonexistent in these countries (Akyol et al., 2006). Through hand hygiene literatures, *Akyol* et al. has reviewed hand washing process in health care from a worldwide perspective and has explored some aspects of hand washing which attracted little attention or needed more attention and finally focused on cultural issues of it. They believe that hand hygiene compliance is poor worldwide among medical personnel while it is widely accepted as the milestone of infection control hospitals, especially in critical care units. They have mentioned that while improving the compliance with hand hygiene recommendations depends on cultural issues or human behaviors, some aspects of hand washing are needed more attention to gain better results in this field, such as hand drying, hand creams and emollients, wearing gloves, rings, wrist watches, bracelets, sleeves, cuffs, fingernails, nail technology and nail polish, having hand tattoos and cultural issues all of which determine hand hygiene behavior (Akyol et al., 2006). *McLaws* et al. have determined different aspects of hand hygiene from the health care workers’ viewpoint. They have conducted their research in two hospital settings in Shiraz, Iran, through eight focus group discussions and six in-depth interviews with ICU and surgical ward nurses, physicians and supporting staff. In their qualitative research, hand hygiene compliance was studied in relation with three themes: personal factors, environmental factors, and the health system. In *McLaws*’ research, being allergic to hand hygiene materials as a personal factor, an emergency situation and heavy workload as environmental factors and beliefs in the role of supervision and obligation as the impact of health system on hand hygiene were the examples of noncompliance of hand washing causes (McLaws et al., 2015); so they are the same as “Insufficient and/or imperfect hand wash” failure causes mentioned in the study (See Table 3). But in comparison, the emergency or the critical situation of patients are categorized in “other factors” group which are related to patients according to ECM, not as an environmental factor. In another research, *Song, Stockwell, Floyd, Short, & Singh* in their retrospective FMEA study have described a systematic process for improving hand hygiene compliance in the Neonatal Intensive Care Unit (NICU) and evaluated its impact on patient’s outcome. They have showed that, overall, hand washing rate has been increased from 50.3% pre intervention (July 2008-September 2008) to 84.0% post intervention (January 2009-September 2011) which resulted in a saving of 11.6 NICU-days and $66,397 hospital charges. Finally, their study has demonstrated the FMEA application to improve hand hygiene and act as a potential cost-effective tool for preventing methicillin-resistant Staphylococcus aureus (MRSA) in hospitals. Besides, *Song* et al. have listed causes for poor hand hygiene compliance in fishbone diagram in four groups: culture/behavior/attitude, supply/environment, work flow/process, and knowledge. It can be seen that some of the determined causes are the same as obtained results in hospitals A and B (See Table 3), such as skin irritation from frequent gel use, lack of knowledge about hand hygiene policy, lack of understanding the importance of hand hygiene, no accountability and lack of signs for reminders (Song, Stockwell, Floyd, Short & Singh, 2013). In this manner, *Su* et al. in their prospective research have evaluated the impact of the International Nosocomial Infection Control Consortium (INICC) Multidimensional Hand Hygiene (HH) Approach in three ICUs in China to analyze hand hygiene compliance factors. They implemented this approach by improving the administrative support, supplies availability, education, reminders in the workplace, process surveillance and performance feedback. Results have indicated that overall hand hygiene compliance increased from 51.5% to 80.1% while several variables were significantly related to poor health hygiene compliance: females vs. males (64% vs. 55%), nurses vs. physicians (64% vs. 57%), among others. Finally, they have concluded that with the INICC multidimensional approach, health hygiene compliance improved significantly (Su et al., 2015).

It should be noted that according to *McLaws* hand hygiene results conducted in Iran and the similarity of the “Insufficient and/or imperfect hand wash” failure as a top high risk failure in both selected hospitals of this research can show that this kind of failure is not only an organizational problem, but also a cultural and national problem in Iran which needs more attention.

### 4.2 Second High Risk Failures

Second high risk failures were “non-consideration of patient caring requirements” in patient delivery process from Operating Room (OR) to ICU in hospital A and “not viewing patient radiography and leave it to another physician” in portable radiography process in hospital B.

One of the most important latent causes for “non-consideration of patient caring requirements” failure in the recovery room in hospital A was lack of continuity approach to patient care process by OR nurses while they often focused on just delivering patients fast to ICUs and pay little attention to the continuity of patient medication or safety requirements during and after the delivering process. It seems that this potential cause can also reinforce the other causes such as lack of information of patient injected drug in the recovery room given to the next nurse who can be ICU nurse. This way, *Heideveld-Chevalking, Calsbeek, Damen, Gooszen, & Wolff* were somehow considered noncompliance of health care guidelines failure while they retrospectively analyzed medical errors which had been recorded in the Hospital Incident Management System (HIMS); the Patient Safety Company database in the Netherlands from July 2009 to July 2012. They presented that from totally 2,563 incidents (1,300 adverse events and 1,263 ‘near-miss’ events) through 67,360 operations in the Radboud University Medical Center, most incidents were reported by anesthesia, OR and recovery nurses (37%) in comparison with ward nurses (31%), physicians (17%), administrative personnel (5%) and others. Besides, these incident causes were classified into five categories which were human (68%), organizational (23%), technical (2%), patient-related (3%) and other factors (4%). According to the reported causes, most incidents were related to not following Standard Operative Procedure (SOPs) (16.2%), human mistake or having forgotten (15.4%) and communication issues (11.5%). *Heideveld-Chevalking* et al. have concluded that while professionals themselves have reported noncompliance with SOPs, human factor was the most important issue and after that communication shortcoming, mistakes and forgetting were important targets for reducing preoperative incidents in the studied hospital. In other words, improving guideline compliance and effective communication are required to improve patient safety in the end (Heideveld-Chevalking, Calsbeek, Damen, Gooszen, & Wolff, 2014).

The most important cause of “not viewing patient’s radiography and leave it to another physician” failure in hospital B is the over crowdedness of the ICU which makes physicians leave their patient graphs viewing to the next shift physician without informing him/her. Moreover, despite the use of Picture Archiving and Communication Systems (PACS), this failure can’t be prevented by alarming or informing to related physician and bedside nurse. In other words, detectability of this decision as a failure is not high enough even by using PACS to prevent from occurring and having a longtime effect for patient. Considering the crucial importance of an effective communication in the delivery of healthcare in the ICU, *Hanis, Georgiou and Westbrook* conducted a systematic review to assess all studies between 1980 and 2010 which consider PACS impact on clinicians’ workflow in the ICU. They found out that all studies have measured some aspects of the PACS introduction time, image availability, image reviewing time taken by a physician, and changes in viewing patterns. Moreover, the impact on clinical decision-making has been assessed in seven studies which were mostly the time impact to image-based clinical action and PACS effect on communication modes reported in five studies. Overly, it seems that PACS makes changes in the communication between clinicians and radiologists, while potential clinicians interpret images independently without any radiology advice. Furthermore, three studies have shown some decrease in the radiologist clinician communication after the PACS introduction and two of them have shown no changes (Hains, Georgiou, & Westbrook, 2012).

### 4.3 Third High Risk Failures

Third high risk failures were “Wrong diagnosis for the patient’s suction need” in suction process in hospital A and “Imperfect/not sterile intubation” in endotracheal/tracheal intubation process in hospital B.

For “wrong diagnosis for patient’s suction need” failure, heavy workload, a novice nurse, alternative nurse, wrong patient triage, and the ambiguous respiratory statue of patients (delivered from ER or OR) are mentioned as this failure causes. On the other hand, “Imperfect/not sterile intubation” failure causes in endotracheal/tracheal intubation were: the rapid turnover of medical staff, suctioning at high speed, suctioning with not sterile gloves, and not having enough sterile gloves and requirements. The severity of having no sterile suction and endotracheal/tracheal intubation severity was as high as the nosocomial infection which even resulted in patient’s death. In developing countries, recent review research on Health Care Associated Infections (HAIs) has shown that about 15% of hospitalizations have resulted in an HAI, which is higher in comparison to estimated rates (4%-10%) in developed countries. The most important causes of high HAIs in developing countries are resource limitations make infection control measures challenging (such as hand hygiene, proper disinfection of medical equipment and surfaces, injection safety and waste management). In addition, some common factors in developing countries can increase the likelihood of respiratory pathogens transmission in health care settings such as over crowdedness of hospital wards, lack of training and fewer programs on the prevention of nosocomial infections (Ndegwa et al., 2014). Moreover, imperfect intubation brings about the re-intubation need which has an effect on ICU stay time (Azarfarin, Ashouri, Totonchi, Bakhshandeh & Yaghoubi, 2014).

### 4.4 FMEA Research in Healthcare

Literature reviews in the field of applying FMEA shows that this method is still new with a few health- related industries and service processes (Chuang, 2010). However, applying systematic quality improvement tools like FMEA is highly recommended in any hospital process (Abbasgholizadeh Rahimi et al., 2013). Most FMEA studies on health care industries have concluded that this method is a simple practical tool for improving healthcare systems. In this research, identifying and analyzing failures and their causes and then suggesting correction actions in two selected ICUs processes show the FMEA’s high capability to identify, measure, prioritize and analyze all potential failure modes in such a complex and critical process, ICU. Besides, the systematic approach of FMEA makes researchers systematically manage medical errors as well and it also permits them to modify their steps according to research objectives. Other FMEA research in healthcare was concluded the effectiveness of FMEA in their way. For example, *Oakalkar and et al*. have used a PFMEA as a proven engineering tool for identifying haemodialysis process requirements, potential failures and causes, evaluating related risk and finally implementing correction actions by reducing occurrence and improving controls. In this study, researchers believe that FMEA approach can cover other hospital activities as well (Ookalkar et al., 2009). *Chiozza and Ponzetti* have shown that the FMEA introduction in laboratory medicine is strongly supported while it can improve patient care safety process and reduce total cost. They have mentioned that FMEA results can be short-lived if the clinical laboratory management doesn’t support continuous safety improvement (Chiozza, & Ponzetti, 2009). In other similar research, *Alonso-Ovies and et* al. conducted a FMEA method to improve ICU process by focusing on human resources only. They analyzed failures related to novice nureses incorporation in the ICU processes and could decrease clinical risks by training programs (Alonso-Ovies et al., 2010). *Al Tehewy and et al*. have also applied FMEA in order to mitigating risks of infusion therapy in a ICU and measured medication errors pre and post of intervention. By showing the pre-intervention medication errors reduction, they have proved FMEA effectiveness in eliminating ICU infusion pricess and improving staff satisfaction (Al Tehewy, El Hosseini, Habil, Abdel Maaboud, & Abdel Rahman, 2015). In another most similar research, *Asefzadeh and et al*. have applied FMEA for eliminating ICU’s clinical errors in one Qazvin hospital in Iran during 2011. They have suggested correction actions in four general categories considering focus groups: 1) training and clinical care improvements 2) shift work scheduling and motivation of nurses 3) hiring experienced, enthusiastic and skillful work force 4) purchase, maintenance, repair and calibration of medical equipment. They have concluded that the ICUs are potentially an attractive area for early adoption of FMEA; While strong, effective leadership and sustained commitment are needed for successful FMEA implementation (Asefzadeh et al., 2013). Not only FMEA is a simple practical tool whose process is systematically defined in details, but it also permit users (FMEA team) to modify their steps according to research objectives and conditions (Welborn, 2010; Sawhney et al., 2010). However, this method can face barriers such as being highly time consuming and costly to be implemented properly.

## 5. Conclusion

Applying of modified PFMEA for improving two selected ICUs’ processes reliability in two different kind of hospitals (governmental and non-governmental) shows that this prospective and systematic method empowers staff to identify, evaluate, prioritize and analyze all potential failure modes and also make them eager to identify their causes, recommend corrective actions and even participate in improving process without feeling blamed by top management. Moreover, by combining FMEA and ECM, team members could easily identify and classify failure causes at the point of health care perspectives.

In other words, identifying and analyzing 18 not accepted failures from 378 potential failures in hospital A and 42 non-accepted ones from 184 failure modes in hospital B, identifying and classifying their causes and then suggesting correction actions based on failure causes in two selected ICUs processes show the FMEA’s high capability to identify and analyze all potential failure modes in such a complex and critical process, ICU. Besides, the systematic approach of FMEA makes researchers systematically manage medical errors as well and it also permits FMEA team to modify their steps according to research objectives.

**Appendix A Table A:** General characteristics of FMEA team members in two studied ICUs

	Hospital A*				Hospital B**			
	
	Position	Education	Experienced year in hospital	ICU	Position	Education	Experienced year in hospital	ICU
1	Chief nursing	Bachelor of midwifery, Master of business administration (MBA)	26	<1	Head nurse of Neurosurgical ICU	Bachelor of nursing	20	11
2	Assistant of chief nursing	Bachelor of nursing	22	<1	Head nurse of General ICU (GICU)	Bachelor of nursing	27	3
3	Educational nurse supervisor	Bachelor of nursing, Master of science in health education	25	18	Head nurse of Post ICU	Bachelor of nursing, Master of science in entrepreneurship	24	5
4	Clinical nurse supervisor	Bachelor of nursing	25	5	Quality Improvement expert charge	Bachelor of nursing, Master of management	19	11
5	Head nurse of Surgical ICU (SICU)	Bachelor of nursing	19	10				
6	Head nurse of Open Heart ICU	Bachelor of nursing	23	18				
7	Head nurse of General ICU (GICU)	Bachelor of nursing	24	20				
8	ICU bedside nurse and Patient safety expert charge	Bachelor of nursing	10	3				

* nongovernmental hospital in Tehran which belongs to some of governmental organization but are managed privately;

** governmental and educational hospital in Tehran which relates to Tehran University of Medical Sciences (TUMS).
